# Hidden and Rare Food Allergens in Pediatric Age

**DOI:** 10.3390/nu15061386

**Published:** 2023-03-13

**Authors:** Leonardo Tomei, Antonella Muraro, Mattia Giovannini, Simona Barni, Giulia Liccioli, Erika Paladini, Lucrezia Sarti, Benedetta Pessina, Isabel Skypala, Elio Novembre, Francesca Mori

**Affiliations:** 1 Allergy Unit, Meyer Children’s Hospital IRCCS, 50139 Florence, Italy; 2 Department of Health Sciences, University of Florence, 50139 Florence, Italy; 3 Food Allergy Referral Centre Department of Mother and Child Health, University of Padua, 35128 Padua, Italy; 4 Department of Allergy & Clinical Immunology, Royal Brompton & Harefield Hospitals, Part of Guys & St Thomas NHS Foundation Trust, London SW3 6LR, UK; 5 Inflammation, Repair & Development Section, National Heart & Lung Institute, Imperial College, London SW7 2BX, UK

**Keywords:** hidden, rare, food allergens, pediatric age, children

## Abstract

In food allergy management, the avoidance of the allergen that caused the reaction plays a fundamental role. Nevertheless, that can be thwarted in case of accidental exposure to a rare or hidden allergen, causing the adoption of a monotonous diet and a consequent reduction in the quality of life of the patient and their family. The identification of a rare and hidden allergen is an important diagnostic challenge, taking into account that a significant proportion of all food reactions is in reality due to them. The aim of the present review is to provide the pediatric allergist an overview of the possible sources of rare and hidden food allergens, taking into consideration the routes of exposure to these potential allergens with the main examples published in the scientific literature and the distinction between types of direct or cross-contamination. The identification of the allergen responsible for the reaction and the provision of a dietary advice customized for the specific individual’s dietary habits is essential to improve quality of life of the familiar nucleus and to reduce the risk of further allergic reactions.

## 1. Introduction

In food allergy management, the avoidance of the allergen that caused the reaction plays a fundamental role [1]. However, even if avoidance is scrupulously carried out, accidental exposure to the allergen is still possible, which can result in serious signs and symptoms, such as anaphylaxis [2]. Accidental exposure and the consequent anxiety often result in a decreased quality of life for the patient and their family. Concern about accidental exposure can lead to the adoption of monotonous diets that are potentially deficient from a nutritional point of view, or of changes in behavior, such as social isolation [3].

A patient can be exposed to a rare or hidden food allergen. As a result, many cases of so-called idiopathic anaphylaxis are potentially due to unidentified allergens as reported in a retrospective study by Añíbarro et al., that showed that 22.4% of food allergic reactions in individuals over 14 years of age were due to hidden allergens [4]. 

**Figure 1:**
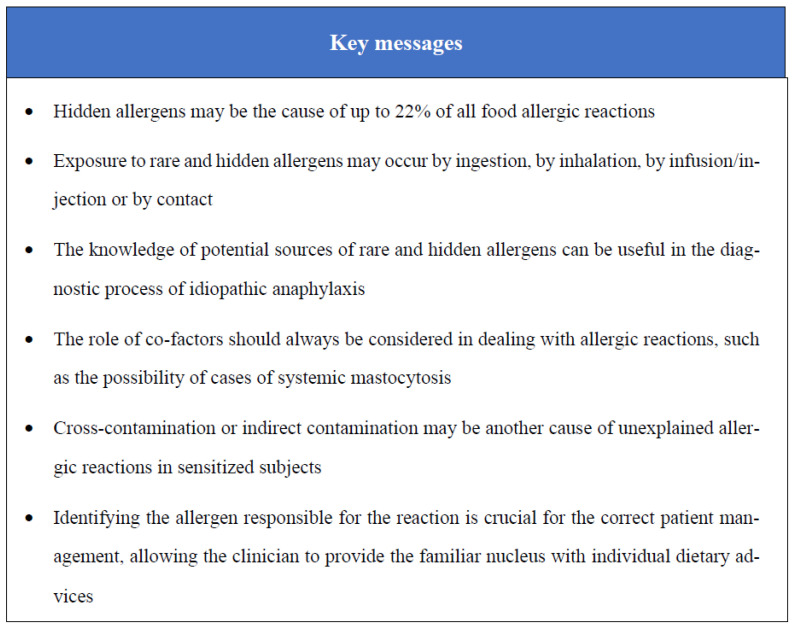
Key messages.

The purpose of this review is to provide the pediatric allergist with an overview of the challenge of the management represented by the avoidance of rare and hidden food allergens, taking into consideration the possible routes of exposure to these potential allergens, with the main examples published in the scientific literature and the distinction between types of direct or cross-contamination. All these elements can be particularly useful to guide the doctor’s choice, based on the risk profile of the individual patient, to enable the balanced management of the allergic child, including food allergy-related labeling and precautionary labeling themes (Figure 1). 

Exposure to allergens may occur for example by contact, by ingestion because it is inside a specific food source or drug, by inhalation, e.g., in outdoor and indoor environments (domestic or non-domestic), or by infusion (i.e., drugs or vaccines) (Table 1). Knowing the triggering sources of potential allergens exposure could help in the prevention and/or prompt recognition of accidental allergen exposure, so that appropriate management can be instigated, thus avoiding progression of severe clinical manifestations.

## 2. Ingestion

The ingestion of hidden food allergens can occur mainly during the intake of products on the market, for example as a consequence of incomplete and/or incorrect labeling [5] or due to the changes in allergic potential during the food processing [6].

The hidden foods can be identified with the use of molecular biology laboratory techniques such as the Enzyme-Linked Immunosorbent Assay (ELISA), which targets a specific protein [7], and the Polymerase Chain Reaction (PCR), which targets a specific nucleic acid [8]. A recent very reliable technique is represented, then, by mass spectrometry [9]. 

### 2.1. Food Additives

Food additives are defined by the Joint Food and Agriculture Organization (FAO)/World Health Organization (WHO) Expert Committee on Food Additives (JECFA) as “substances added to food to maintain or improve its safety, freshness, taste, texture, or appearance” [10]. In contrast to the general opinion, the prevalence of reactions to food additives is less than 1% in adults, but it is thought to be up to 2% in children [11]. 

### 2.2. Colorants

Carmine (cochineal, E120) is a naturally derived red dye derived from dried female insects of the species *Dactylopius coccus Costa*, and it has been linked with anaphylactic reactions with an IgE-mediated mechanism [12]. Additionally, in two studies carmine was associated with flares of atopic eczema in children. Machler and Jacob [13] described the case of a four-year-old girl with recurrent episodes of generalized dermatitis. The patch-test result was positive to carmine and other substances, but the post patch evaluation revealed relevant sources of carmine in many foods retrospectively connected to the onset of flares. Dietary avoidance of carmine resulted in a remarkable clinical improvement at 18 months follow-up, with a single episode of flare of dermatitis that developed within 5 hours of drinking a fruit punch containing carmine red. Catli et al. [14] compared patch-test positivity between a group of children with atopic eczema and a group of healthy children. A positive patch-test to carmine was significantly higher in the atopic eczema group as was the consumption of food containing this additive. 

Likewise, hypersensitivity to annatto (another naturally derived colorant) was described in two case reports. The first [15] concerned a two-year-old female who developed recurrent hives after the ingestion of food containing annatto. However, given the young age of the child, no further in vivo or in vitro tests were performed. The second report [16] concerned a five-year-old girl, who presented with two episodes of generalized urticaria after the ingestion of commercially prepared foods with annatto as a common ingredient. Prick-to-prick testing with annatto was positive. 

Tartrazine, a synthetic food dye, has been linked with cutaneous adverse reactions, although these reactions are not often confirmed by rigorous testing. In two studies [17,18], double-blind placebo-controlled challenges with tartrazine were performed in children with suspected hypersensitivity to this additive. Adverse reactions to tartrazine were confirmed in 3/19 patients in the first study and in 1/12 patients in the second one. 

### 2.3. Preservatives

Sulfites are chemical products used as additives, e.g., in food and pharmaceutical products for their antioxidant properties and also because they prevent browning. Although sulfite reactions are more common in adults, bronchospasm after oral challenge testing was also described in asthmatic children [19,20]. Furthermore, a more recent case report described a five-year old female with a history of gastrointestinal and cutaneous clinical manifestations after the ingestion of several different preserved foods and oral drugs containing sodium metabisulphite (SMB) [21]. Based on the clinical history and by the patch test result, a diagnosis of SMB allergy was made. After the start of a diet free from this additive, the child had a sudden improvement of her gastrointestinal and cutaneous signs and symptoms. 

Sodium benzoate (E211), naturally present in some foods such as cinnamon, is also used as a preservative and can be found, e.g., in commercially prepared foods (e.g., beverages, especially soft drinks), cosmetics, and pharmaceutical products. One report cited the case of a 12-year-old boy who developed cheilitis after the ingestion of cola and canned baked beans containing that additive [22]. Additionally, sodium benzoate was identified as the culprit of cutaneous adverse drug reactions in 10 children that reported maculopapular eruptions or urticaria after the intake of amoxicillin plus clavulanic acid suspension, probably with a non-immunologic mechanism [23].

### 2.4. Thickening Agents

Pectin, a thickening agent, has been identified as a possible hidden allergen, especially in patients sensitized to cashew and pistachio. In the first of two case reports [24], a three-year-old male developed an anaphylactic reaction after drinking a strawberry fruit smoothie, whereas in the second [25] a five-year old female presented an anaphylaxis after the ingestion of jellybeans; pectin was listed as an ingredient in both food products. Both children had a positive skin prick test (SPT) to the additive, but other potential food allergens were negative. Interestingly, both patients had positive specific serum IgE for cashew and pistachio. Concomitant allergy to pectin and to these tree nuts has been reported in adult subjects [26,27,28], but the mechanism underlying this phenomenon has not been clarified.

Anaphylaxis to carboxymethylcellulose, another thickening agent, has been reported. In adults this has been noted after the use of parenteral corticosteroid preparations [29,30] and barium sulfate contrast medium [31]. This additive, used, e.g., in pharmaceutical preparations, wound dressings, cosmetics, and foods, has also been linked to anaphylactic reactions in a 14-year-old female [32]. The girl experienced several episodes of anaphylaxis requiring intramuscular adrenaline after eating an ice lolly and a half-frozen beverage, both containing carboxymethylcellulose. SPTs to this additive were positive and an Oral Food Challenge (OFC) with the ice lolly resulted in the development of hives and dyspnoea, although the consumption of the same ice cream without carboxymethylcellulose made by the manufacturer did not induce any reactions. After the avoidance of this allergen, at six months follow-up the child had no anaphylactic reactions, except for an episode induced by a noodle product containing carboxymethylcellulose that she had eaten by mistake.

### 2.5. Flavorings

Spices, such as fenugreek and pepper, frequently found in both homemade and commercially prepared food, can act as hidden allergens [33]. In many cases, spice allergy is believed to be a secondary effect after primary sensitization with inhalant allergens [34]. 

A case report [35] described a 17-month-old male that had an anaphylactic reaction requiring intramuscular epinephrine after eating venison prepared with a marinade containing various spices. SPTs were positive to black and cayenne pepper and specific IgE to cayenne pepper was also positive, whereas SPTs and specific IgE to the other ingredients in the spice mix and the meal were negative. 

Fenugreek (*Trigonella foenum-graecum*), an herb that belongs to the *Leguminosae* family, can be an ingredient in tea, curry, and chutney. It has been identified as the culprit of an anaphylactic reaction in a 14-year-old male who developed hives, chest constriction, abdominal pain, and emesis after the consumption of a spread containing fenugreek, lemon, garlic, and cilantro [36]. After this episode, the boy avoided fenugreek, but consumed and tolerated the other ingredients. On evaluation, SPTs with a solution containing ground fenugreek were positive and he also had a high specific IgE level to fenugreek. In order to verify that this was a non-irritant concentration, the same solution was tested in four healthy individuals without reactions. As it is a member of the *Leguminosae* family, fenugreek has considerable cross-reactivity with peanut proteins [37]. Fæste et al. [38] analyzed the sera from patients that reported anaphylaxis to legume-containing foods including 11 children. In most cases, the peanut IgE levels were higher or equal to the fenugreek IgE levels. 

Several clinical studies including children have focused on mustard allergy [39]. As a result of its wide use to enhance flavor, mustard can be a hidden allergen in various foods, including some baby and toddler pre-packaged foods. In the areas where its consumption is higher, as in France, mustard is a more common allergen [40]. 

Herbs belonging to the *Lamiaceae* family have been linked with allergic reactions. A 13-year-old boy developed two episodes of angioedema after the consumption of chicken meat containing oregano and after drinking sage tea [41]. A prick-to-prick test with oregano, sage, and mint was positive only for mint. However, the OFC with sage induced angioedema after 25 minutes.

### 2.6. Cow’s Milk Proteins

Cow’s milk allergy is one of the most common food allergies in infants and young children and cross-contamination with cow’s milk proteins can cause severe reactions in highly allergic individuals. Levin et al. described the case of a nine-month-old boy with cow’s milk allergy that developed anaphylaxis and respiratory arrest after the ingestion of a dry-powder soy formula [42]. Samples of the formula were analyzed for milk, egg white, and peanut proteins, and ß-lactoglobulin was found both in the mixed formula and in the dry powder. 

Milk proteins have also been found in oral polio vaccine (Sabin vaccine) [43]. Parisi et al. described a case series of four patients with milk protein allergy that developed acute allergic reactions after vaccination. Every child had positive SPTs to both milk protein and oral polio vaccine and levels of milk protein and Sabin vaccine-specific IgE were increased. In addition, an ELISA using specific rabbit antiserum detected α-lactalbumin in the Sabin vaccine. 

Even probiotics can be a source of hidden allergens, as demonstrated by a Spanish study that reported the presence of cow’s milk proteins in 10/11 of compounds tested and the presence of hen’s egg proteins in 3/11 of compounds tested [44]. Additionally, a French study showed that 2/3 of probiotics used widely in France contain significant amounts of β-lattoglobulin [45]. The papers also reported the cases of two children that had developed anaphylaxis after the ingestion of two different probiotic compounds, and both patients had increased milk-specific IgE levels. 

### 2.7. Hidden Allergens in Other Food Products

Antibiotic residues present in food can be a cause of allergic reactions of unknown origin, as described in a 10-year-old girl who had an anaphylaxis after the ingestion of a blueberry pie that was found to contain a non-ß-lactam antibiotic after bacterial growth inhibition testing [46]. Given that streptomycin is commonly used to treat bacterial infections of fruit, cutaneous allergy testing for streptomycin was performed, and an intradermal skin test with a dilution of 1:1000 resulted positive. The child also showed neck urticaria a few minutes after the test and a late-phase reaction with generalized urticaria and inspiratory stridor three hours later. The girl had never been treated with streptomycin in the past.

The use of latex gloves by food handlers is a potential route of inadvertent exposure to latex allergens, as reported in a 10-year-old boy that developed an anaphylactic reaction 10 minutes after eating a cream-filled doughnut [47]. SPTs, prick-to-prick tests, and serum-specific IgE for each ingredient used during the preparation of the doughnut were negative, but results for SPTs using a commercial latex extract were positive. A double-blind food challenge was also performed, and the child developed urticaria, conjunctivitis, and lip oedema after eating of the doughnut prepared using latex gloves, while he did not develop any clinical manifestation with the doughnut prepared without using them. 

Domestic mites are an important allergen source in respiratory allergic diseases, but they can also be the cause of severe allergic signs and symptoms immediately after eating foods prepared with wheat flour contaminated with various species of them [48]. These reactions, named as “Pancake syndrome”, are usually caused by foods prepared with wheat flour, especially pancakes, but also, e.g., pizza, pasta, and bread. Given that cooked foods are able to induce the clinical manifestations, the allergens involved in these reactions are supposed to be thermoresistant. 

Allergen of *Anisakis simplex*, a parasitic nematode contained in a large proportion of commercial fish and seafood, has been described as the cause of relapsing acute urticaria in children and adolescents not sensitized to Ascaris or fish [49]. Some *Anisakis simplex* allergens are highly resistant to heat and freezing [50]. Sensitization may occur even in populations with low consumption of raw or undercooked fish. 

Sweets could be implicated in children’s allergic reactions, e.g., with cow’s milk, egg, nut, or fruit allergies, but they can also contain uncommon allergens, as described in the case of a 21-month-old boy who developed perioral urticaria with lip oedema, abdominal pain, vomiting, and generalized urticaria after the ingestion of candies [51]. At the investigation, a prick-to-prick test was positive to one of the brands of candies ingested. The SPTs to each of the components was positive only to a vegetable protein, identified by the manufacturer as potato peel protein.

### 2.8. Plant Food Allergens

Non-specific Lipid Transfer Proteins (nsLTPs) are a cause of IgE-mediated food-allergic reaction in children and in adults. Allergy to nsLTP occurs predominantly in the Mediterranean area, but it is less common in northern and central Europe [52,53,54,55]. These proteins are widely expressed throughout the plant kingdom and there is a high degree of cross reactivity between nsLTP allergens from botanically unrelated fruits and vegetables [56]. Although fruits remain the most frequent food involved, allergens from this family have also been identified in, e.g., nuts, vegetables, cereals, legumes, and seeds [54], and thus nsLTP allergy should be considered in the diagnostic process after reactions to one of these foods. These allergens are thermostable and hence may also be present as hidden allergens in composite dishes. Reactions to nsLTP are frequently linked to cofactors, especially exercise and intake of non-steroidal anti-inflammatory drugs (NSAIDs), while other cofactors such as fasting, alcohol, antacids, temperature, oestrogens, other drugs (e.g., angiotensin-converting enzyme inhibitors, β-blockers, lipid-lowering drugs), stress, and fatigue could be implicated [54]. 

Gibberellin-regulated proteins (GRPs) are allergens recently identified in plant-derived food allergies. These proteins have been found in peach (Pru p 7), apricot (Pru m 7), orange (Cit s 7), and pomegranate (Pun g 7), and they have heat and digestion stability similar to nsLTP, being able to cause systemic and often severe reactions, even in children [57]. Patients allergic to GRPs can react to fruits belonging not only to the *Rosaceae* family (such as peach, apricot, apples, strawberry, and cherries) but also to the *Rutaceae* family (such as lemon and oranges) [58]. Allergy to GRPs should be taken into account during the diagnosis after systemic reactions to fruit, especially in case of Pru p 3 sIgE negativity.

### 2.9. Uncommon Food Allergens

#### 2.9.1. Alpha-Gal Syndrome

Uncommon food allergens include α-1,3-galactose (alpha-gal), which may cause both an immediate hypersensitivity reaction to drugs containing that disaccharide (e.g., cetuximab) [59], and a delayed reaction due to the ingestion of meat from non-primate mammals. It is still uncertain what drives IgE production for alpha-gal, but epidemiological data from the USA showed that sensitization to alpha-gal could occur following a bite from the *Amblyomma americanum* tick, and in other continents from the *Ixodes* species. Alpha-Gal Syndrome (AGS) has unusual characteristics. Clinical manifestations appear about 3–6 hours after the meal, do not occur after every ingestion and the onset of the condition could start years after sensitization and involve food previously tolerated [60]. 

#### 2.9.2. Cat-Pork Syndrome

Another condition that can cause reactions after the eating of red meat is cat-pork syndrome. This condition may affect cat allergic individuals, sensitized via the respiratory tract to cat albumin Fel d 2, who then develop allergic signs and symptoms after the consumption of pork or beef meat. This occurs because IgE against Fel d 2 can cross-react with porcine albumin (Sus s 1) and beef albumin (Bos d 6) [61]. Pork serum albumin is heat-labile, so well-cooked pork seems more likely to be tolerated, whereas fresh, dried, or smoked preparations of meat are more likely to elicit reactions. Cat-pork syndrome is more common in adults, but some pediatric cases were reported [61,62], even after consuming beef intestines [63]. Additionally, a case of FDEIA (Food-Dependent Exercise-Induced Anaphylaxis) was described in a 13-year-old boy that developed urticaria and dyspnoea during intense exercise after ingestion of pork [64]. 

#### 2.9.3. Bird-Egg Syndrome

Poultry meat allergy may also occur as a secondary food allergy resulting from cross-reactivity, in the context of bird-egg syndrome. This condition is due to sensitization to serum albumins present in many tissues including bird muscle tissue and egg yolk (Gal d 5) [65,66]. Primary sensitization to serum albumin may occur via the respiratory tract through exposure to pet birds such as pigeons, budgerigars, and parrots in adults, or within the context of egg allergy in early childhood [67]. While cross-sensitization to poultry meat is common in patients with bird-egg syndrome, clinical reactions after meat ingestion appear to be rare because of the heat-lability of serum albumins [68].

#### 2.9.4. Novel Allergic Cross-Recognitions

The increasing popularity and accessibility of exotic foods could lead to the discovery of new allergens and novel allergic cross-reactivities. Ballardini et al. [69] reported the case of a 13-year-old boy with severe allergy to chicken meat who developed an anaphylactic reaction after the ingestion of crocodile meat. Molecular analysis led to the identification of a crocodile α-parvalbumin, with extensive sequence homology to chicken α-parvalbumin. Another case report [70] described anaphylaxis in a nine-year-old boy with a fish allergy following the ingestion of crocodile meat. In this case, cross-reactivity between parvalbumins of both species was identified as the cause of the reaction. Additionally, in vitro cross-recognition between cod parvalbumin and frog parvalbumin was described [71]. Despite SPTs performed with recombinant frog parvalbumin being positive in fish-allergic patients, to the best of our knowledge, no case of reaction to frog meat had been described in children. 

In some cases, the chemical reactions that take place during the cooking process could lead to the production of compounds that may cause allergic clinical manifestations in some children, as described in the case report of a five-year-old boy [72] who developed urticaria after eating homemade caramel made by cooking condensed sweet milk for two hours. The child regularly ate milk (fresh, condensed, or condensed sweetened) without any problem. At the investigations, SPTs with caramel resulted positive and the boy was instructed to avoid caramel. In this case, the non-enzymatic browning reactions, such as Maillard reaction, of sweetened milk could have led to the production of compounds that could be responsible for the clinical manifestations. 

## 3. Inhalation

In some cases, allergic reactions can occur when dietary proteins are inhaled. Examples of this dynamic can be the inhalation of steam coming from the cooking of foods (e.g., fish, shellfish, and milk), or due to the release of tiny amounts of powdered food into the environment following their crushing and/or grinding (e.g., milk). This type of reaction is usually mild, but, in rare cases, can also cause more severe reactions.

A food allergen that has to be carefully considered with reference to the risk of inhalation is lupine. In fact, pulverized lupine can be present in products not destined for food such as, for example, fertilizer for plants. Its presence as an inhaled hidden allergen in rural areas can give rise to unexpected allergic reactions [73]. 

Lactose is an inactive ingredient common in many pharmaceutical products that is used, e.g., to improve the stability of active substances in medicines, including drugs to treat allergic conditions such as asthma, even in the acute phase. Although medical grade lactose is usually free of milk protein, it can convey milk protein as described in the case of a nine-year-old boy that developed a refractory asthma exacerbation after the use of lactose-containing dry powder inhalers [74]. 

Even toys can be a source of hidden allergens, as described in the case report of a nine-year-old boy with multiple food allergies including wheat and corn, who developed a severe anaphylactic reaction that needed epinephrine administration after contact with a toy fingerprint powder [75]. It was evaluated with the ELISA, which was strongly positive for gluten, likely coming from wheat flour.

## 4. Infusion/Injection

The contamination of lactose with milk proteins is also found in corticosteroids to be taken parenterally as a methylprednisolone powder and solvent for solution for injection and can therefore be responsible for serious systemic reactions once infused in subjects with milk allergy [76].

On the internet it is possible to find a list of drugs containing lactose, although these lists cannot be considered exhaustive as they are subject to continuous updating. A safer alternative is to look for the presence, among the excipients, of a particular potential food allergen in the Ministerial Technical Data Sheet (SPC) of each single drug.

There may be many potential contaminants to look for beyond milk proteins. For example, some benzylpenicillin products may contain lecithin contaminated by soy proteins and used as an excipient [77]. 

In the scientific literature, cases of anaphylaxis after intravenous administration of paracetamol with previous tolerance of this drug taken orally are also described [78]. In these patients a specific sensitization to mannitol, as an excipient in numerous drugs, can be demonstrated.

In rare cases an infusion reaction due to a food allergen has been described after introduction of the food protein by blood transfusion from a subject who, before the donation, had consumed certain foods such as, for example, peanut [79].

## 5. Contact

The contact between the allergen and the skin can cause urticaria or other skin signs or symptoms, especially in the point where the allergen touches the skin. Severe contact reactions between skin and allergens are rare, but one example of a skin reaction from contact with hidden foods is sensitization to hydrolyzed wheat proteins due to the use of facial cleansers, which may precede and/or be associated with reactions to the intake of wheat [80]. Wheat proteins may also be present in some types of moldable wheat flour-based materials, usually used in play by children [81].

The situation is different when the skin barrier is disrupted, e.g., atopic dermatitis, when allergen penetration is facilitated, therefore enhancing the likelihood of sensitization and/or allergic contact reactions to foods and simple chemicals.

Hidden allergens can also be found in topical preparations, as reported for polyvinylpyrrolidone (PVP), an excipient present in medical products such as tablets and ophthalmic solutions but also in personal care items and in foods (E1201) [82]. A paper reported the case of a 15-year-old girl that developed anaphylaxis after the administration of corticosteroid eye drops containing PVP. At allergy work-up, prick-by-prick to PVP and to eye drops that caused the reaction test resulted positive, while they resulted negative to other eye drops without the excipient that the girl used without any reactions. Anaphylaxis to PVP was also described in a nine-year-old boy that developed two systemic reactions after the administration of povidone-iodine (PVP-I) for impetigo contagious [83]. Interestingly, the child had no reaction after previously using PVP-I as an antiseptic agent, so the lesioned skin due to impetigo contagious could have played a role in the developing of reactions. 

**Table 1:** Potential sources of rare and hidden allergens.

Allergen	Potential Source of Exposure	Reference
**Ingestion**		
**Food Additives**		
CARMINE (E120)	e.g., several commercially prepared foods including cheese, fruit, and vegetables preparations, processed fish and fishery products, jams, meat products, soups, sauces, desserts, and drinks	[13,14]
ANNATTO (E160B)	e.g., several commercially prepared foods including cheese, processed fish and fishery products, desserts, jams, meat products, soup, and sauces	[15,16]
TARTRAZINE (E102)	e.g., several commercially prepared foods including cheese, canned and bottled fruits and vegetables, soups, processed fish and fishery products, sauces, and non-alcoholic drinks	[17,18]
SULFITES (E220-228)	e.g., several foods including dried fruits, fruit juices, dried potatoes, desserts, processed fish and fishery products and alcoholic and non-alcoholic drinks	[21]
SODIUM BENZOATE (E211)	e.g., foods, pharmaceutical preparations, cosmetics	[22,23]
PECTIN (E440)	e.g., thickening agent in several food including candies	[24,25]
CARBOXYMETHYLCELLULOSE (E466)	e.g., pharmaceutical preparations, wound dressings, cosmetics, and foods such as chocolate products, ice creams, frozen cakes, instant pasta, condiments	[32]
**Spices**		
PEPPER	e.g., seasoning in several foods	[35]
FENUGREEK	e.g., seasoning in several foods	[36]
MUSTARD	e.g., seasoning in several foods including baby foods and commercial foods for toddlers	[39,40]
*LAMIACEE* FAMILY	e.g., spice mixes and curry powder	[41]
**Cow’s Milk Proteins**		
BETA-LACTOGLOBULIN	e.g., alternatives milk formulas	[42]
ALPHA-LACTALBUMIN	e.g., polio Sabin vaccine	[43]
COW’S MILK PROTEINS	e.g., probiotics	[44,45]
**Other Food Products**		
STREPTOMYCIN	e.g., foods containing antibiotic residues	[46]
LATEX ALLERGENS	e.g., foods processed with latex gloves	[47]
DOMESTIC MITES	e.g., foods prepared by wheat flour	[48]
*ANISAKIS SIMPLEX*	e.g., commercial fishes and cephalopods	[49,50]
POTATO PEEL PROTEIN	e.g., candies	[51]
**Meat Allergens**		
ALPHA-GAL SYNDROME	e.g., meat from non-primate mammals, intravenous drugs (such as cetuximab)	[59,60]
CAT PORK SYNDROME	e.g., red meat	[61,62,63,64]
BIRD-EGG SYNDROME	e.g., poultry meat	[65,66]
**Uncommon Food Allergens**		
PARVALBUMINS	e.g., crocodile meat	[69,70]
**Inhalation**		
LUPIN	e.g., fertilizer for plant	[73]
COW’S MILK PROTEIN	e.g., dry powder inhalers	[74]
WHEAT FLOUR	e.g., toy fingerprint kit	[75]
**Infusion**		
COW’S MILK PROTEIN	e.g., methylprednisolone powder and solvent for solution for injection	[76]
SOY PROTEIN	e.g., benzylpenicillin	[77]
MANNITOL	e.g., paracetamol	[78]
PEANUT ALLERGENS	e.g., blood transfusion	[79]
**Contact**		
WHEAT PROTEINS	e.g., facial cleanser, mouldable plastic materials	[80,81]
POLYVINILPIRROLIDONE (E1201)	e.g., corticosteroid eye drops	[83]

## 6. The Role of Co-Factors

In some cases, a sole exposure to an allergen is not able to induce anaphylaxis [84], but it could occur only in association with certain co-factors, such as exercise, alcohol, menses, psychological stress, and NSAIDs. Data from different studies and registries report that cofactors play a role in about 30% of all anaphylactic reactions in adults [85]. 

In the case of Food-Dependent Exercise Induced Anaphylaxis (FDEIA), the reaction appears only when food is eaten in association with physical activity, either pre- or post-exercise. FDEIA reactions can be related to both plant and animal proteins but the most common foods reported include wheat, in particular omega-5-gliadin, LTP allergens, shellfish, and nuts [86]. NSAID intake has been reported to be present in up to 22% of food-induced severe anaphylactic reactions. Foods more frequently involved are wheat, plant foods and shellfish [87]. Finally, infectious diseases can act as cofactors, with a reported relevance from anaphylaxis registries of 2.5–3% in children [85].

## 7. Mastocytosis

Due to the similarity in clinical manifestations, systemic mastocytosis can be potentially misdiagnosed as food-induced anaphylaxis [88]. Some scoring systems, based on combined clinical and laboratory parameters, have been proposed in order to discern these two conditions [89,90,91]. 

## 8. Cross-Contamination (Indirect Contamination)

Cross-contamination (or indirect contamination) represents an additional risk for patients with food allergies and occurs when a certain allergen is accidentally localized in another food: for example, by using an inadequately cleansed container that previously contained another food [92]; on the surface of an object, e.g., kitchen utensils (e.g., forks, spoons, knives), pans, dishes or cooking surfaces that have not been properly cleaned before preparing and/or cooking food [93]; in saliva: food allergens can be transmitted by people and home pets [94]. Indeed, everything that is introduced into the oral cavity can represent a possible source of cross-contamination.

Cross-contamination represents a particular management problem in the context of food allergies, as the allergen is hidden, and its intake is unpredictable [95]. Moreover, especially in adolescence, new risks of contamination arise due to the general lower attention to health in this age and the tendency to share food with peers [96].

The pediatrician can give specific advice to deal with these problems and limit the risk of indirect contamination [97]. Other suggestions that the pediatrician can give are offered by the systematic guidelines for the management of food allergy in schools, published by the Centers for Disease Control and Prevention (CDC) [98] and the British Anaphylaxis Campaign [99].

## 9. Diagnosis

Identifying a rare or hidden allergen is an essential diagnostic challenge when approaching an allergic reaction of unclear cause, for example, idiopathic anaphylaxis. Collecting a detailed clinical history is the first and more important step, allowing the identification of possible sources of allergens. Thereafter, a diagnostic workup that comprises in vivo and in vitro tests could lead to the identification of the sensitization of the subject [88]. Although it is difficult, identifying the allergen responsible for the reaction is crucial for the correct patient management, allowing the clinician to provide the family with individual dietary advices [100].

## 10. Management

The primary therapeutic strategy for managing a food allergy consists of strictly avoiding the allergen and treating accidental exposure with medications [101]. However, novel therapeutical approaches are under investigation for the treatment of food allergies; the ultimate goal of these therapies is to induce permanent immune tolerance, defined as the unresponsiveness to allergens either in natural exposure settings or in vivo challenges [102]. Therapeutic approaches to food allergies can be classified as food allergen-specific immunotherapy and food allergen non-specific immunotherapy [103]. Allergen-specific immunotherapy (AIT) consists in exposing the subject to gradually increased doses of the allergen and includes mainly oral (OIT), subcutaneous (SCIT), sublingual (SLIT), and epicutaneous (EPIT) immunotherapy [104]. Food AIT aims to downregulate the allergic immune response, taking as targets, e.g., IgE, cytokines, cells, or genes [105]. Between biological drugs, omalizumab is the most widely studied molecule for treating food allergies, used both as monotherapy and adjunctive therapy in food AIT [106]. Despite findings showing encouraging results, food AIT and other experimental therapy need further research through international, high-quality, and extensive works to reach a consensus on the clinical application of these strategies [104].

## 11. Conclusions

The personalization of advice regarding rare and hidden allergen avoidance needs to be considered based on the individual patient’s food triggers and characteristics and the consequent risk level. The provision of individual dietary advice is a powerful weapon to reduce the risk of reaction to food and ensure the quality of life of families of young patients with food allergy [107].

However, avoiding trace amounts is difficult to maintain in the long term. The psychological consequences of food exclusion can be sometimes even more serious than the risk of food allergy reactions, and lead to neophobia, or fear of new foods [108], and consequent adoption of monotonous and potentially nutritionally-deficient diets [109]. In recent years, an active treatment of food allergies is increasingly being undertaken [110], based on desensitization—oral immunotherapy to the specific food, which is thus gradually reintroduced starting from exceedingly small quantities. The reintroduction of even tiny amounts of food makes it possible to enlarge the diet considerably and therefore to improve the quality of life of the child and the family.

## Abbreviation

AGS = alpha-gal syndrome; AIT = allergen-specific immunotherapy; CDC = Centers for Disease Control and Prevention; ELISA = enzyme-linked immunosorbent assay; EPIT = epicutaneous immunotherapy; FAO = Food and Agriculture Organization; FDEIA = food-dependent exercise-induced anaphylaxis; GRPs = Gibberellin-regulated proteins; JECFA = Joint FAO/WHO Expert Committee on Food Additives; NSAIDs = non-steroidal anti-inflammatory drugs; nsLTPs = non-specific Lipid Transfer Proteins; OFC = oral food challenge; OIT = oral immunotherapy; PCR = polymerase chain reaction; PVP = polyvinylpyrrolidone; PVP-I = povidone-iodine; SCIT = subcutaneous immunotherapy; SLIT = sublingual immunotherapy; SMB = Sodium metabisulphite; SPC = ministerial technical data sheet; SPT = skin prick test; WHO = World Health Organization.

## Data Availability

Not applicable.

## References

[1] Cafarotti A., Giovannini M., Begìn P., Brough H.A., Arasi S. (2023). Management of IgE-mediated food allergy in the 21st century. Clin. Exp. allergy J. Br. Soc. Allergy Clin. Immunol..

[2] Muraro A., Tropeano A., Giovannini M. (2022). Allergen immunotherapy for food allergy: Evidence and outlook. Allergol. Sel..

[3] Mori F., Giovannini M., Barni S., Jiménez-Saiz R., Munblit D., Biagioni B., Liccioli G., Sarti L., Liotti L., Ricci S. (2021). Oral Immunotherapy for Food-Allergic Children: A Pro-Con Debate. Front. Immunol..

[4] Añíbarro B., Seoane F.J., Múgica M.V. (2007). Involvement of hidden allergens in food allergic reactions. J. Investig. Allergol. Clin. Immunol..

[5] Taylor S.L., Baumert J.L. (2015). Worldwide food allergy labeling and detection of allergens in processed foods. Chem. Immunol. Allergy.

[6] Pekar J., Ret D., Untersmayr E. (2018). Stability of allergens. Mol. Immunol..

[7] Do A.B., Khuda S.E., Sharma G.M. (2018). Undeclared Food Allergens and Gluten in Commercial Food Products Analyzed by ELISA. J. AOAC Int..

[8] Di Girolamo F., Muraca M., Mazzina O., Lante I., Dahdah L. (2015). Proteomic applications in food allergy: Food allergenomics. Curr. Opin. Allergy Clin. Immunol..

[9] Cristina L., Elena A., Davide C., Marzia G., Lucia D., Cristiano G., Marco A., Carlo R., Laura C., Gabriella G.M. (2016). Validation of a mass spectrometry-based method for milk traces detection in baked food. Food Chem..

[10] WHO (2018). Food Additives.

[11] Laura A., Arianna G., Francesca C., Carlo C., Carla M., Giampaolo R. (2019). Hypersensitivity reactions to food and drug additives: Problem or myth?. Acta Biomed..

[12] Wüthrich B., Kägi M.K., Stücker W. (1997). Anaphylactic reactions to ingested carmine (E120). Allergy Eur. J. Allergy Clin. Immunol..

[13] Machler B.C., Jacob S.E. (2018). Carmine Red: A Potentially Overlooked Allergen in Children. Dermatitis.

[14] Catli G., Bostanci I., Ozmen S., Dibek Misirlioglu E., Duman H., Ertan U. (2015). Is Patch Testing with Food Additives Useful in Children with Atopic Eczema?. Pediatr. Dermatol..

[15] Myles I.A., Beakes D. (2009). An Allergy to Goldfish? Highlighting Labeling Laws for Food Additives. World Allergy Organ. J..

[16] Ramsey N.B., Tuano KT S., Davis C.M., Dillard K., Hanson C. (2016). Annatto seed hypersensitivity in a pediatric patient. Ann. Allergy Asthma Immunol..

[17] Wilson N., Scott A. (1989). A double-blind assessment of additive intolerance in children using a 12 day challenge period at home. Clin. Exp. Allergy.

[18] Devlin J., David T.J. (1992). Tartrazine in atopic eczema. Arch. Dis. Child..

[19] Towns S.J., Mellis C.M. (1984). Role of acetyl salicylic acid and sodium metabisulfite in chronic childhood asthma. Pediatrics.

[20] Boner A., Guarise A., Vallone G., Fornari A., Piacentini F., Sette L. (1990). Metabisulfite oral challenge: Incidence of adverse responses in chronic childhood asthma and its relationship with bronchial hyperreactivity. J. Allergy Clin. Immunol..

[21] Vitaliti G., Guglielmo F., Giunta L., Pavone P., Falsaperla R. (2015). Sodium metabisulphite allergy with multiple food and drug hypersensitivities in a five-year-old child: A case report and literature review. Allergol. Immunopathol..

[22] Jacob S.E., Hill H., Lucero H., Nedorost S. (2016). Benzoate allergy in children—From foods to personal hygiene products. Pediatr. Dermatol..

[23] Mori F., Barni S., Pucci N., Rossi M.E., De Martino M., Novembre E. (2012). Cutaneous Adverse Reactions to Amoxicillin-Clavulanic Acid Suspension in Children: The Role of Sodium Benzoate. Curr. Drug Saf..

[24] Ferdman R.M., Ong P.Y., Church J.A. (2006). Pectin anaphylaxis and possible association with cashew allergy. Ann. Allergy Asthma Immunol..

[25] Baker M.G., Saf S., Tsuang A., Nowak-Wegrzyn A. (2018). Hidden allergens in food allergy. Ann. Allergy Asthma Immunol..

[26] Kraut A., Peng Z., Becker A.B., Warren C.P. (1992). Christmas candy maker’s asthma; IgG4-mediated pectin allergy. Chest.

[27] Räsänen L., Mäkinen-Kiljunen S., Harvima R.J. (1998). Pectin and cashew nut allergy: Cross-reacting allergens?. Allergy Eur. J. Allergy Clin. Immunol..

[28] Hernández-García E., de las Heras M., Bartolomé B., Compés E., Sastre J., Cuesta J. (2004). Anaphylaxis caused by the pectin component of barium sulphate suspension*1. J. Allergy Clin. Immunol..

[29] Bigliardi P.L., Izakovic J., Weber J.M., Bircher A.J. (2003). Anaphylaxis to the carbohydrate carboxymethylcellulose in parenteral corticosteroid preparations. Dermatology.

[30] Laisuan W., Wongsa C., Dchapaphapeaktak N., Tongdee M., Chatmapanrangsee J., Rerkpattanapipat T. (2012). Anaphylaxis following intralesional triamcinolone acetonide (Kenacort) injection. Asia Pac. Allergy.

[31] Dumond P., Franck P., Morisset M., Laudy J.S., Kanny G., A Moneret-Vautrin D. (2009). Pre-lethal anaphylaxis to carboxymethylcellulose confirmed by identification of specific IgE—Review of the literature. Eur. Ann. Allergy Clin. Immunol..

[32] Ohnishi A., Hashimoto K., Ozono E., Sasaki M., Sakamoto A., Tashiro K., Moriuchi H. (2019). Anaphylaxis to carboxymethylcellulose: Add food additives to the list of elicitors. Pediatrics.

[33] Chen J.L., Bahna S.L. (2011). Spice allergy. Ann. Allergy Asthma. Immunol..

[34] Schöll I., Jensen-Jarolim E. (2004). Allergenic potency of spices: Hot, medium hot, or very hot. Int. Arch. Allergy Immunol..

[35] Gimenez L., Zacharisen M. (2011). Severe pepper allergy in a young child. Wis. Med. J..

[36] Joseph N.I., Slavin E., Peppers B.P., Hostoffer R.W. (2018). Fenugreek Anaphylaxis in a Pediatric Patient. Allergy Rhinol..

[37] Fæste C.K., Christians U., Egaas E., Jonscher K.R. (2010). Characterization of potential allergens in fenugreek (Trigonella foenum-graecum) using patient sera and MS-based proteomic analysis. J. Proteomics.

[38] Fæste C.K., Namork E., Lindvik H. (2009). Allergenicity and antigenicity of fenugreek (Trigonella foenum-graecum) proteins in foods. J. Allergy Clin. Immunol..

[39] Sharma A., Verma A.K., Gupta R.K., Neelabh, Dwivedi P.D. (2019). A Comprehensive Review on Mustard-Induced Allergy and Implications for Human Health. Clin. Rev. Allergy Immunol..

[40] Dalal I., Binson I., Reifen R., Amitai Z., Shohat T., Rahmani S., Levine A., Ballin A., Somekh E. (2002). Food allergy is a matter of geography after all: Sesame as a major cause of severe IgE-mediated food allergic reactions among infants and young children in Israel. Allergy Eur. J. Allergy Clin. Immunol..

[41] Yazici S., Nacaroǧlu H.T., Erdem S.B., Karaman S., Can D. (2018). Angioedema due to lamiaceae allergy. Iran. J. Allergy Asthma Immunol..

[42] Levin M.E., Motala C., Lopata A.L. (2005). Anaphylaxis in a milk-allergic child after ingestion of soy formula cross-contaminated with cow’s milk protein. Pediatrics.

[43] Parisi CA S., Smaldini P.L., Gervasoni M.E., Maspero J.F., Docena G.H. (2013). Hypersensitivity reactions to the Sabin vaccine in children with cow’s milk allergy. Clin. Exp. Allergy.

[44] Martín-Muñoz M.F., Fortuni M., Caminoa M., Belver T., Quirce S., Caballero T. (2012). Anaphylactic reaction to probiotics: Cow’s milk and hen’s egg allergens in probiotic compounds. Pediatr. Allergy Immunol..

[45] Lee T.-T.T., Morisset M., Astier C., Moneret-Vautrin D.-A., Cordebar V., Beaudouin E., Codreanu F., Bihain B.E., Kanny G. (2007). Contamination of probiotic preparations with milk allergens can cause anaphylaxis in children with cow’s milk allergy. J. Allergy Clin. Immunol..

[46] Graham F., Paradis L., Bégin P., Paradis J., Babin Y., Roches A.D. (2014). Risk of allergic reaction and sensitization to antibiotics in foods. Ann. Allergy Asthma Immunol..

[47] Bernardini R., Novembre E., Lombardi E., Pucci N., Marcucci F., Vierucci A. (2002). Anaphylaxis to latex after ingestion of a cream-filled doughnut contaminated with latex. J. Allergy Clin. Immunol..

[48] Bódi B., Cojanu C., Gorbatovschi V., Ureche C. (2018). Pancake syndrome—Oral mite anaphylaxis. Alergologia.

[49] Falcão H., Lunet N., Neves E., Iglésias I., Barros H. (2008). Anisakis simplex as a risk factor for relapsing acute urticaria: A case-control study. J. Epidemiol. Community Health.

[50] Pontone M., Giovannini M., Barni S., Mori F., Venturini E., Galli L., Valleriani C., Vecillas L.D.L., Sackesen C., Lopata A.L. (2023). IgE-mediated Anisakis allergy in children. Allergol. Immunopathol..

[51] Martín-Muñoz M.F., Diaz-Perales A., Cannabal J., Quirce S. (2017). Anaphylaxis to hidden potato allergens in a peach and egg allergic boy. Eur. Ann. Allergy Clin. Immunol..

[52] Betancor D., Gomez-Lopez A., Villalobos-Vilda C., Nuñez-Borque E., Fernández-Bravo S., Gozalo M.D.L.H., Pastor-Vargas C., Esteban V., Cuesta-Herranz J. (2021). Ltp allergy follow-up study: Development of allergy to new plant foods 10 years later. Nutrients.

[53] Asero R., Piantanida M., Pinter E., Pravettoni V. (2018). The clinical relevance of lipid transfer protein. Clin. Exp. Allergy.

[54] Skypala I.J., Bartra J., Ebo D.G., Faber M.A., Fernández-Rivas M., Gomez F., Luengo O., Till S.J., Asero R., Barber D. (2021). The diagnosis and management of allergic reactions in patients sensitized to non-specific lipid transfer proteins. Allergy Eur. J. Allergy Clin. Immunol..

[55] Vereda A., van Hage M., Ahlstedt S., Ibañez M.D., Cuesta-Herranz J., van Odijk J., Wickman M., Sampson H.A. (2011). Peanut allergy: Clinical and immunologic differences among patients from 3 different geographic regions. J. Allergy Clin. Immunol..

[56] Asero R., Mistrello G., Roncarolo D., Amato S., Caldironi G., Barocci F., Van Ree R. (2002). Immunological cross-reactivity between lipid transfer proteins from botanically unrelated plant-derived foods: A clinical study. Allergy Eur. J. Allergy Clin. Immunol..

[57] Biagioni B., Tomei L., Valleriani C., Liccioli G., Barni S., Sarti L., Citera F., Giovannini M., Mori F. (2021). Allergy to Gibberellin-Regulated Proteins (Peamaclein) in Children. Int. Arch. Allergy Immunol..

[58] Inomata N. (2020). Gibberellin-regulated protein allergy: Clinical features and cross-reactivity. Allergol. Int..

[59] Caglayan-Sozmen S., Santoro A., Cipriani F., Mastrorilli C., Ricci G., Caffarelli C. (2019). Hazardous Medications in Children with Egg, Red Meat, Gelatin, Fish, and Cow’s Milk Allergy. Medicina.

[60] Saretta F., Giovannini M., Mori F., Arasi S., Liotti L., Pecoraro L., Barni S., Castagnoli R., Mastrorilli C., Novembre E. (2021). Alpha-Gal Syndrome in Children: Peculiarities of a “Tick-Borne” Allergic Disease. Front. Pediatr..

[61] Wilson J.M., Platts-Mills T.A.E. (2019). Red meat allergy in children and adults. Curr. Opin. Allergy Clin. Immunol..

[62] Yamada S., Matsubara K., Chinuki Y., Hori M., Masaki T. (2019). Early childhood-onset pork-cat syndrome due to sensitization by both cats and dogs—a case report. Arerugi.

[63] Sagawa N., Inomata N., Suzuki K., Sano S., Watanabe Y., Aihara M. (2021). Pork-cat syndrome caused by ingestion of beef intestines in an 8-year-old child. Allergol. Int..

[64] Shiratsuki R., Chinuki Y., Fukushiro S., Morita E. (2020). A case of pork-cat syndrome that developed as food-dependent exercise-induced anaphylaxis. Acta Derm. Venereol..

[65] Mandallaz M.M., De Weck A.L., Dahinden C.A. (1988). Bird-egg syndrome. Cross-reactivity between bird antigens and egg-yolk livetins in IgE-mediated hypersensitivity. Int. Arch. Allergy Appl. Immunol..

[66] Szepfalusi Z., Ebner C., Pandjaitan R., Orlicek F., Scheiner O., Boltznitulescu G., Kraft D., Ebner H. (1994). Egg yolk α-livetin (chicken serum albumin) is a cross-reactive allergen in the bird-egg syndrome. J. Allergy Clin. Immunol..

[67] Hemmer W., Klug C., Swoboda I. (2016). Update on the bird-egg syndrome and genuine poultry meat allergy. Allergo J. Int..

[68] Quirce S., Maranon F., Umpierrez A., Heras M.D.L., Fernandez-Caldas E., Sastre J. (2001). Chicken serum albumin (Gal d 5*) is a partially heat-labile inhalant and food allergen implicated in the bird-egg syndrome. Allergy Eur. J. Allergy Clin. Immunol..

[69] Ballardini N., Nopp A., Hamsten C., Vetander M., Melén E., Nilsson C., Ollert M., Flohr C., Kuehn A., van Hage M. (2017). Anaphylactic reactions to novel foods: Case report of a child with severe crocodile meat allergy. Pediatrics.

[70] Haroun-Díaz E., Blanca-López N., de la Torre M.V., Ruano F.J., Álvarez M.L.S., Horrillo M.L., Bartolomé B., Blanca M., Díez G.C. (2018). Severe anaphylaxis due to crocodile-meat allergy exhibiting wide cross-reactivity with fish allergens. J. Allergy Clin. Immunol. Pract..

[71] Hilger C., Thill L., Grigioni F., Lehners C., Falagiani P., Ferrara A., Romano C., Stevens W., Hentges F. (2004). IgE-antibodies of fish allergic patients cross-react with frog parvalbumin. Allergy Eur. J. Allergy Clin. Immunol..

[72] Christopoulou G., Tziotou M., Psomiadou M., Tzeli K., Karantoumanis D., Kostoudi S., Zisaki V., Georgiou K., Roumpedaki E., Douladiris N. (2015). Allergy in products of non enzymatic browning reactions—A case report. Allergy.

[73] Novembre E., Moriondo M., Bernardini R., Azzari C., Rossi M.E., Vierucci A. (1999). Lupin allergy in a child. J. Allergy Clin. Immunol..

[74] Robles J., Motheral L. (2014). Hypersensitivity Reaction After Inhalation of a Lactose-Containing Dry Powder Inhaler. J. Pediatr. Pharmacol. Ther..

[75] Taylor S.L., Baumert J.L., Boudreau-Romano S.M. (2017). Allergic reaction from fingerprint kit attributable to unlabeled gluten, probable wheat flour. J. Allergy Clin. Immunol. Pract..

[76] Savvatianos S., Giavi S., Stefanaki E., Siragakis G., Manousakis E., Papadopoulos N.G. (2011). Cow’s milk allergy as a cause of anaphylaxis to systemic corticosteroids. Allergy.

[77] Barni S., Mori F., Pantano S., Novembre E. (2015). Adverse reaction to benzathine benzylpenicillin due to soy allergy: A case report. J. Med. Case Rep..

[78] Jain S.S., Green S., Rose M. (2015). Anaphylaxis following intravenous paracetamol: The problem is the solution. Anaesth. Intensive Care.

[79] Jacobs J.F., Baumert J.L., Brons P.P., Joosten I., Koppelman S.J., van Pampus E.C. (2011). Anaphylaxis from passive transfer of peanut allergen in a blood product. New Engl. J. Med..

[80] Fukutomi Y., Taniguchi M., Nakamura H., Akiyama K. (2014). Epidemiological link between wheat allergy and exposure to hydrolyzed wheat protein in facial soap. Allergy Eur. J. Allergy Clin. Immunol..

[81] Ponda P., Sicherer S. (2005). An Allergic Reaction to Play-Doh in a Child With Wheat Hypersensitivity. J. Allergy Clin. Immunol..

[82] Liccioli G., Mori F., Barni S., Pucci N., Novembre E. (2018). Anaphylaxis to polyvinylpyrrolidone in eye drops administered to an adolescent. J. Investig. Allergol. Clin. Immunol..

[83] Yoshida K., Sakurai Y., Kawahara S., Takeda T., Ishikawa T., Murakami T., Yoshioka A. (2008). Anaphylaxis to polyvinylpyrrolidone in povidone-iodine for impetigo contagiosum in a boy with atopic dermatitis. Int. Arch. Allergy Immunol..

[84] Barni S., Mori F., Giovannini M., de Luca M., Novembre E. (2019). In situ simulation in the management of anaphylaxis in a pediatric emergency department. Intern. Emerg. Med..

[85] Wölbing F., Fischer J., Köberle M., Kaesler S., Biedermann T. (2013). About the role and underlying mechanisms of cofactors in anaphylaxis. Allergy Eur. J. Allergy Clin. Immunol..

[86] Foong R.X., Giovannini M., du Toit G. (2019). Food-dependent exercise-induced anaphylaxis. Curr. Opin. Allergy Clin. Immunol..

[87] Sánchez-López J., Araujo G., Cardona V., García-Moral A., Casas-Saucedo R., Guilarte M., Torres M.J., Doña I., Picado C., Pascal M. (2021). Food-dependent NSAID-induced hypersensitivity (FDNIH) reactions: Unraveling the clinical features and risk factors. Allergy Eur. J. Allergy Clin. Immunol..

[88] Bilò M.B., Martini M., Tontini C., Mohamed O.E., Krishna M.T. (2019). Idiopathic anaphylaxis. Clin. Exp. Allergy.

[89] Carter M.C., Desai A., Komarow H.D., Bai Y., Clayton S.T., Clark A.S., Ruiz-Esteves K.N., Long L.M., Cantave D., Wilson T.M. (2018). A distinct biomolecular profile identifies monoclonal mast cell disorders in patients with idiopathic anaphylaxis. J. Allergy Clin. Immunol..

[90] Gülen T., Hägglund H., Sander B., Dahlén B., Nilsson G. (2014). The presence of mast cell clonality in patients with unexplained anaphylaxis. Clin. Exp. Allergy.

[91] Alvarez-Twose I., González-De-Olano D., Sánchez-Muñoz L., Matito A., Jara-Acevedo M., Teodosio C., García-Montero A., Morgado J., Orfao A., Escribano L. (2012). Validation of the REMA score for predicting mast cell clonality and systemic mastocytosis in patients with systemic mast cell activation symptoms. Int. Arch. Allergy Immunol..

[92] Boyano-Martínez T., García-Ara C., Pedrosa M., Díaz-Pena J.M., Quirce S. (2009). Accidental allergic reactions in children allergic to cow’s milk proteins. J. Allergy Clin. Immunol..

[93] Furlong T.J., Desimone J., Sicherer S.H. (2001). Peanut and tree nut allergic reactions in restaurants and other food establishments. J. Allergy Clin. Immunol..

[94] Kim J.S., Sicherer S.H. (2011). Living with Food Allergy: Allergen Avoidance. Pediatr. Clin. North Am..

[95] Taylor S.L., Baumert J.L. (2010). Cross-contamination of foods and implications for food allergic patients. Curr. Allergy Asthma Rep..

[96] Cummings A.J., Knibb R.C., King R.M., Lucas J.S. (2010). The psychosocial impact of food allergy and food hypersensitivity in children, adolescents and their families: A review. Allergy Eur. J. Allergy Clin. Immunol..

[97] Pistiner M., LeBovidge J., Bantock L., James L., Harada L. (2013). Living Confidently with Food Allergy Handbook: A Guide for Parents and Families.

[98] Centers for Disease Control and Prevention (2013). Voluntary Guidelines for Managing Food Allergies in Schools and Early Care and Education Programs.

[99] Anaphylaxis Campaign Model Policy for Allergy Management at School. https://www.anaphylaxis.org.uk/wp-content/uploads/2021/10/Model-Policy-for-allergy-management-at-school.pdf.

[100] Barni S., Liccioli G., Sarti L., Giovannini M., Novembre E., Mori F. (2020). Immunoglobulin E (IgE)-mediated food allergy in children: Epidemiology, pathogenesis, diagnosis, prevention, and management. Medicina.

[101] Muraro A., Werfel T., Hoffmann-Sommergruber K., Roberts G., Beyer K., Bindslev-Jensen C., Cardona V., Dubois A., Dutoit G., Eigenmann P. (2014). EAACI Food Allergy and Anaphylaxis Guidelines: Diagnosis and management of food allergy. Allergy Eur. J. Allergy Clin. Immunol..

[102] Alvaro-Lozano M., Akdis C.A., Akdis M., Alviani C., Angier E., Arasi S., Arzt-Gradwohl L., Barber D., Bazire R., Cavkaytar O. (2020). EAACI Allergen Immunotherapy User’s Guide. Pediatr. allergy Immunol. Off. Publ. Eur. Soc. Pediatr. Allergy Immunol..

[103] Dantzer J.A., Wood R.A. (2019). Next-Generation Approaches for the Treatment of Food Allergy. Curr. Allergy Asthma Rep..

[104] Pajno G.B., Fernandez-Rivas M., Arasi S., Roberts G., Akdis C.A., Alvaro-Lozano M., Beyer K., Bindslev-Jensen C., Burks W., Ebisawa M. (2018). EAACI Guidelines on allergen immunotherapy: IgE-mediated food allergy. Allergy Eur. J. Allergy Clin. Immunol..

[105] Passanisi S., Caminiti L., Zirilli G., Lombardo F., Crisafulli G., Aversa T., Pajno G.B. (2021). Expert Opinion on Biological Therapy Biologics in food allergy: Up-to-date Biologics in food allergy: Up-to-date. Expert Opin. Biol. Ther..

[106] Worm M., Francuzik W., Dölle-Bierke S., Alexiou A. (2021). Use of biologics in food allergy management. Allergol. Sel..

[107] Logan K., Du Toit G., Giovannini M., Turcanu V., Lack G. (2020). Pediatric Allergic Diseases, Food Allergy, and Oral Tolerance. Annu. Rev. Cell Dev. Biol..

[108] Feng C., Kim J.H. (2019). Beyond Avoidance: The Psychosocial Impact of Food Allergies. Clin. Rev. Allergy Immunol..

[109] Meyer R., De Koker C., Dziubak R., Venter C., Dominguez-Ortega G., Cutts R., Yerlett N., Skrapak A.-K., Fox A., Shah N. (2014). Malnutrition in children with food allergies in the UK. J. Hum. Nutr. Diet..

[110] Buyuktiryaki B., Masini M., Mori F., Barni S., Liccioli G., Sarti L., Lodi L., Giovannini M., du Toit G., Lopata A.L. (2021). IgE-Mediated Fish Allergy in Children. Medicina.

